# Morphology of glandular trichome and lignin-based structure for its function

**DOI:** 10.1016/j.fmre.2025.02.014

**Published:** 2025-02-28

**Authors:** Ning Hao, Takehiro Kamiya, Tao Wu

**Affiliations:** a College of Horticulture/Yuelu Mountain Laboratory of Hunan Province, Hunan Agricultural University, Changsha 410128, China; b Graduate School of Agricultural and Life Sciences, University of Tokyo, Tokyo 113-8657, Japan

**Keywords:** Glandular trichome, Morphology, Cell wall structure, Specialized compounds, Storage

## Abstract

Glandular trichomes play critical roles in plant defense, pollination, and environmental adaptation through the synthesis, storage, and secretion of a diverse range of compounds, including specialized metabolites and inorganic compounds. They are also referred to as a “bio-factory” owing to their significant commercial value in industries. The morphology of glandular trichomes significantly affects their capacity to synthesize and store these compounds. Additionally, glandular trichomes develop a lignin-based structure for storing compounds, known as the neck strip, which was first found in cucumber and compartmentalized the substances they produced. This perspective explores the diversity of trichome morphology across different plant species and outlines the cellular structures that facilitate the storage of compounds. Furthermore, we discuss the mechanisms underlying the morphology formation and lignin-based structure in glandular trichomes. These insights may pave the way to innovative strategies for enhancing plant growth and boosting industrial metabolite production.

## 1 Introduction

Glandular trichomes originate from the plant epidermis and are usually multicellular, which can produce and secrete different types of specialized compounds, including essential oils, pigments, terpenes, and alkaloids [1]. These compounds hold commercial importance in industries, particularly in fragrances and pharmaceutical industries. A notable example is *Artemisia annua*, whose glandular trichomes yield artemisinin, a crucial antimalarial drug [2]. Similarly, lavender is extensively cultivated for its glandular trichome-produced essential oils [3]. Beyond their economic significance, glandular trichomes are integral to plant survival strategies, aiding adaptation to environmental stresses through the release of protective chemicals [[4], [5], [6]]. This adaptive function highlights the evolutionary significance of these structures in enhancing plant resilience. For example, tomatoes have four types of glandular trichomes on the stem and leaf with distinct mophologies [5]. In wild tomatoes (*Solanum habrochaites*), type VII glandular trichomes mainly produce sesquiterpenes that have strong anti-herbivore activity and are potential natural pesticides [7]. However, the cultivated tomato (*Solanum lycopersicum*) develops type I and VI trichomes that produce acyl sugars and terpenoids [8,9].

The morphology of glandular trichomes plays important roles in the production of compounds, such as the number and size of gland cells [10], as well as their storage space in gland cells [11]. Furthermore, to prevent the leakage of these compounds back into other cells, some glandular trichomes develop a lignin-based structure that acts as a barrier [12], ensuring that the compounds remain within the gland cells. Owing to these morphological and structural characteristics of glandular trichomes, they can form an efficient system to perform as a factory and enhance the tolerance of plants to various stresses. Therefore, this perspective aimed to explore the biology of glandular trichomes by investigating their morphology and the molecular mechanisms behind their biosynthetic capabilities.

## 2 Morphology of glandular trichomes in storage capacity and secretion of the compounds

**Fig. 1:**
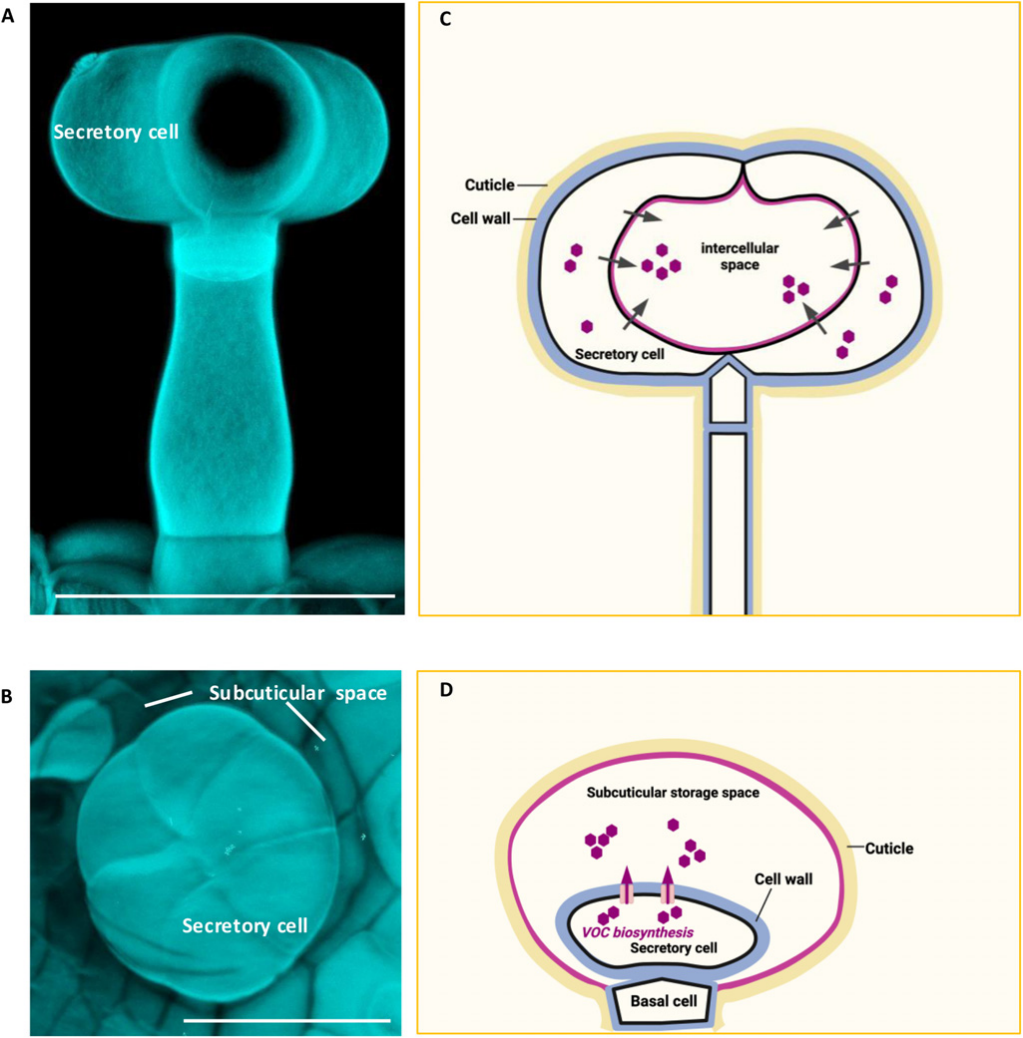
**The morphology of glandular trichomes for compound storage.** (A, B) Confocal microscopy analysis of tomato and peppermint leaf trichome. (A) Type VI glandular trichome on the leaf of tomato. (B) Peltate trichome on the leaf of peppermint. (C, D) The examples of glandular trichome with a storage cavity. The compounds will be stored in the intercellular space in tomato (C), while the subcuticular space in peppermint (D). A, B are stained by calcofluor white M2R and observed under Leica Confocal microscope at 405 nm excitation and emission at 430–470 nm. C, D are modified from reference 11 and 16, respectively. (Scale bars, 50 μm).

The morphology of glandular trichomes in various plant species has been well documented [9,13]. Generally, these trichomes can be classified into two main types: capitate and peltate trichomes. Both types share common structural components such as gland cells, stalk cells, and basal cells [13]. However, peltate trichomes are distinct in having a greater number of gland cells compared to capitate trichomes [13]. Additionally, peltate trichomes possess a subcuticular/intercellular cavity within gland cells [13]. The morphological differences result in the synthesis and storage of distinct types of compounds [14]. Capitate trichomes have limited storage capacity and primarily synthesize the nonvolatile or low-volatility compounds [13,14]. Then, these compounds will be secreted to the tip of the trichome, as observed in type I and type IV trichomes of tomatoes and tobacco [13,14]. In contrast, tomato type VI glandular trichomes are classified as peltate trichomes. These trichomes consist of four gland cells separated by intercellular space (Fig. 1A, 1C). The hydrophobic metabolites secreted by the gland cells are stored in this intercellular space, which can undergo expansion as the trichomes develop [15]. However, the exact composition of the cell wall materials for lining the intercellular space in such glandular trichomes remains unclear [15]. For some certain Lamiaceae plants, like peppermint, the mature stage of peltate trichomes is characterized by multiple secretory cells enveloped by a subcuticular space. The synthesized compounds, for example, some volatile organic compounds will be transported to this space and stored until biotic attack [16] (Fig. 1B, 1D).

Understanding the intricate regulatory mechanisms governing the morphology of glandular trichomes, particularly in model systems like tomatoes, is crucial for comprehending their roles in the formation, storage, and secretion of metabolic products. The identification of key transcription factors such as HD-ZIP (Homeodomain-Leucine zipper) family gene *Wo* (*Woolly*), MYB-like family genes *GCR1* and *2* (*GLAND CELL REPRESSOR 1* and *2*), and bHLH (BASIC HELIX-LOOP-HELIX) family gene *MYC1*, highlights the complexity of genetic networks that orchestrate trichome differentiation and gland formation [8,17,18]. *Wo* determines trichome differentiation in a concentration-dependent manner [17]. High *Wo* concentration will enhance the expression of *SlWox3b* (*WUSCHEL-RELATED HOMEOBOX 3b*) to repress peltate trichome differentiation through antagonistic effects on the expression of the target gene LFS (LEAFLESS) [17]. *GCR1* and *2* have functions at the early stage of trichome development when the tip cells differentiate into glands. Notably, the knockout of *GCR1* and *2* results in more non-glandular trichomes converting into glandular trichomes and increases the gland cell number of type VI and VII glandular trichome [18]. Furthermore, *GCR1* and *2* can regulate LFS to repress the gland formation [18]. *SlMYC1* plays a pivotal role in the development of type VI glandular trichomes in tomatoes, exerting its influence during both the initiation and maturation stages [8]. By elucidating these pathways, researchers can potentially manipulate trichome morphology and enhance the production of desirable metabolites through targeted genetic modifications or environmental interventions, thereby contributing to advances in agricultural biotechnology and natural product discovery.

## 3 Lignin-based structure for storage of the compounds in glandular trichomes

**Fig. 2:**
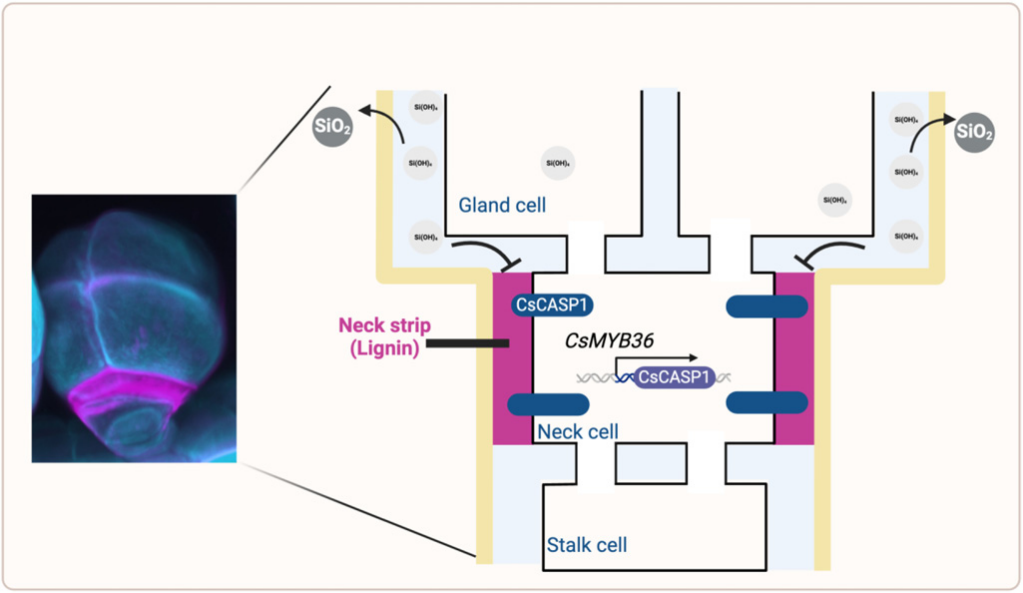
**Lignin-based structure in cucumber glandular trichomes for silica polymerization**. Silicic acid is transported to the cytosol of the gland cell and will be transported to the apoplastic space. In the presence of a neck strip, silicic acid will accumulate in the apoplastic space of the gland cell and move to the trichome surface, thus proceeding with silica polymerization. The formation of the neck strip relies on the localization of CsCASP1 on the plasma membrane of the neck cell and the transcription regulation of *CsMYB36*. The illustration is modified from Reference [12].

Glandular trichomes play pivotal roles in plant evolution, particularly regarding their ability to adapt to diverse environments by producing specific compounds [19]. However, they can synthesize and store compounds of varying types and sizes, which may be related to the diversity of their structures and morphology. In some plants, besides the intercellular/subcuticular space, as shown in Fig. 2, compounds are also stored in the apoplastic area of the glandular trichome by the apoplastic barrier, such as the glandular trichome in some cucurbits [12].

A recent study on cucumbers demonstrated that a novel lignin-based structure, the neck strip, functions as an apoplastic barrier to control the movement of compounds within the glandular trichomes (Fig. 2). Cucumber glandular trichomes contain four to eight divided gland cells to form the glandular head, and a short uniseriate stalk with 3–4 cells [20], which is specifically divided into the neck cell (the intermediate cell of the stalk of a uniseriate, glandular trichome) [21], stalk cell, and basal cell (lowermost cell of a trichome) [21]. The glandular trichome (also referred to as bloom trichome) on the cucumber fruit surface is important for synthesizing the bloom, which is mainly composed of silica and shows a white powder phenotype [22]. The presence of a neck strip in the neck cell in the cucumber glandular trichome prevents the flow of silicic acid from the apoplastic space in the gland cells back to the stalk cells. In addition, it contributes to the accumulation of silicic acid in the gland cells, thereby facilitating the polymerization process of silica, a major component of bloom [12]. The absence of a neck strip will lead to the defect in silicic acid accumulation in the apoplastic space of gland cells and failed silica polymerization process [12]. The observation of the neck strip in glandular trichomes particularly in peltate trichomes across different plant species, including Cucurbitaceae and Lamiaceae, has led to significant insights into the functional adaptations of these structures [12]. Neck strip serves as an apoplastic barrier, which is essential for the efficient storage of various secondary metabolites within the trichome's secretory cavities.

The formation of neck strip in cucumbers is regulated by the CsMYB36-CsCASP1 module. CsCASP1 is a homologue of CASP1 (CASPARIAN STRIP MEMBRANE PROTEIN) in Arabidopsis, a scaffold protein that recruits lignin polymerization-related proteins for Casparian strip formation [23]. In cucumber glandular trichomes, CsCASP1 is a target of *CsMYB36* and its localization is essential for the neck strip formation [12]. Casparian strip and neck strip are two similar lignin-based structures and share a common regulatory mechanism [12,24]. However, they exhibit distinct molecular pathways attributed to their unique biological roles and deposition patterns [12,24]. In the case of the neck strip, lignin is deposited between the plasma membrane of the neck cell and the cuticle [12]. Unlike the localized deposition observed in the Casparian strip, which is located at the confined location in the endodermal cells [24], lignin in the neck strip spreads across the entire lateral surface of the neck cell [12]. These differences suggest variations in the mechanisms underlying lignin deposition and molecular processes, with the notable exception of the MYB36-CASP1 module. Consequently, transcriptome analysis of these two structures could discover some novel genes for neck strip formation and development.

Overall, by identifying and characterizing the morphology and structure in glandular trichomes, researchers can better understand the intricate mechanisms by which these natural cell factories are optimized for their specific biosynthetic roles. Furthermore, the discovery of lignin-based modifications in the form of neck strips underscores the remarkable diversity and complexity of cell wall engineering in plants. The continued research into the molecular and cellular basis of trichome function will shed light on the evolutionary innovations that have shaped the vast array of trichome types found in the plant kingdom. In addition, due to their capacity to produce valuable secondary metabolites in bioengineering and agricultural research, understanding the regulatory mechanisms that govern trichome formation and metabolite synthesis could provide valuable insights for optimizing the “bio-factory” for industrial applications.

## Declaration of competing interest

The authors declare that they have no conflicts of interest in this work.
